# Improving microgrid hosting capacity: A two-stage BONMIN solver-based framework for battery storage allocation and operational energy management strategy

**DOI:** 10.1371/journal.pone.0323525

**Published:** 2025-05-16

**Authors:** Ziad M. Ali

**Affiliations:** Electrical Engineering Department, College of Engineering, Prince Sattam Bin Abdulaziz University, Wadi Addawaser, Saudi Arabia; Institute of Aviation Engineering and Technology, EGYPT

## Abstract

The growing concerns over fossil fuel dependency, environmental impacts, and escalating energy expenses highlight the critical importance of enhancing energy system efficiency. This study presents a dual-phase optimization approach for improving grid-connected microgrid (μG) operations, focusing on Sodium-Sulfur (NaS) and Sodium Nickel Chloride (Na-NiCl₂) battery storage systems. The problem was structured as a mixed-integer nonlinear programming (MINLP) model and resolved using GAMS software with its embedded open-source BONMIN solver. The initial phase establishes optimal battery storage system (BSS) allocation methods to optimize renewable energy source (RES) self-consumption (SC), increase hosting capacity (HC), and minimize operational expenses. Building on these results, the second phase develops optimal microgrid operational strategies to reduce total operating costs further. The research evaluates five scenarios with incrementally increasing the number of BSSs, ranging from one to five units. Through this systematic analysis, the work demonstrates that both the quantity and type of BSS units significantly impact μG operating costs. The most efficient configuration emerged in Case 3, where three Na-NiCl₂ BSS units achieved a 32.35% reduction in operating costs. Additionally, the integration of BSS demonstrated notable improvements in both HC and SC rates.

## 1. Introduction

The global climate crisis is fundamentally driven by fossil fuel consumption, which accounts for over 90% of worldwide carbon emissions through their complete lifecycle - from extraction and processing to final combustion [1,2]. Achieving zero carbon emissions by reducing/eliminating coal, oil, and gas dependency has become essential for climate stabilization and environmental preservation [3]. This imperative directly aligns with sustainability development goals (SDG) such as climate action (SDG#13) and affordable and clean energy (SDG#7) while supporting sustainable cities and communities (SDG#11). Developed nations have positioned the transition toward sustainable, green economies at the forefront of their environmental policies [4]. This fundamental transformation encompasses reducing fossil fuel dependence and minimizing ecological impacts through strategically integrating renewable energy sources (RESs) [5]. The momentum toward sustainability is evidenced by widespread net zero emission commitments, with numerous countries and regions setting ambitious targets for 2050 [6,7]. This global paradigm shift from fossil fuel dependence to RES adoption represents a crucial pathway in addressing the mounting challenges of climate change [8]. The transition addresses environmental concerns and promotes energy security, economic resilience, and technological innovation in the renewable energy sector.

In this context, the accelerated integration of RESs has become instrumental in addressing climate change and enhancing environmental sustainability through various mechanisms [9]. Primarily, clean energy alternatives such as solar and wind power significantly reduce greenhouse gas emissions, vital in mitigating global warming [10]. Additionally, adopting RESs lowers air pollution, improving air quality and health outcomes. The decentralized nature of many renewable technologies also enhances energy security and system resilience by minimizing reliance on centralized power grids, which are often vulnerable to disruptions [11]. Furthermore, the expansion of the renewable energy sector fosters innovation, creates employment opportunities, and drives economic development, collectively supporting a sustainable transition to a greener future [12]. A critical factor in advancing renewable energy adoption is increasing the self-consumption rate (SCR) of RESs, which denotes the proportion of renewable energy generated that is directly utilized on-site [13]. This metric underscores clean energy sources’ efficient and sustainable use [14]. Power systems progressively prioritize SCR to pursue greater economic viability for RESs in electricity generation. This approach emphasizes utilizing renewable energy as it is generated, with any surplus directed to distribution or transmission grids when production exceeds immediate demand. Such strategies aim to optimize operational costs while maximizing the utility of renewable energy [15,16]. This efficient management of electricity grids effectively balances energy production and consumption, contributing to a more sustainable energy system [17]. Further, enhancing SCR rates and the hosting capacity (HC) of RESs has emerged as a pivotal focus in modern active power systems. However, factors such as the intermittent nature of renewable energy and geographical constraints can challenge the consistent supply of electricity [18]. Battery storage systems (BSSs) have proven to be transformative technologies in this context, bolstering system reliability, improving resilience to disruptions, and facilitating greater integration of RESs into grids [19]. BSSs play an essential role by improving power quality (PQ), increasing system reliability and HC of RESs, and mitigating uncertainties associated with renewable generation. Additionally, they reduce peak-time energy imports by supplying peak loads, lowering operational costs, and enhancing electrical networks’ economic and technical performance [20,21].

In the literature, microgrid (μG) systems are recognized for their ability to integrate various distributed generators (DGs), including RESs, BSSs, and multiple hybrid electrical loads [22]. This integration is pivotal in elevating PQ and reliability, reducing transmission losses, improving energy efficiency, and mitigating environmental pollution. However, during peak load periods, transformer overloading can occur due to the limited capacity of the main transformer. While the conventional solution involves expanding transformer capacity, this approach faces challenges such as limited device availability and a low return on investment. To address this issue, shifting peak-time loads to off-peak periods is critical for alleviating transformer load rates [23].

Optimal placement and sizing of BSS, along with the effective coordination of DGs, BSS, and the main grid based on climatic conditions and load demands, are essential for improving the reliability of the power supply. This coordination not only enhances the technical performance of the entire system but also has significant economic and operational implications. Consequently, the study of optimal BSS allocation is of paramount importance. Recent research efforts have focused on evaluating the optimal allocation of energy storage systems (ESSs) and optimizing the power scheduling of DGs, BSS, and the main grid to enhance both the technical and economic performance of μG systems. For instance, Zhang et al. [24] propose a methodology to achieve optimal BES allocation in weak grids to improve system voltage and frequency stability while enhancing reliability. Their approach utilizes the adaptive grey wolf optimization (AGWO) algorithm to identify BESS’s optimal capacity and location within the grid. Validation of their results includes comparisons with other optimization techniques, such as grey wolf optimization (GWO), beluga whale optimization (BWO), and sparrow search algorithm (SSA). Similarly, Inaolaji et al. [25] present a mixed-integer linear programming (MILP) model designed for the optimal sizing and placement of BSS to achieve peak shaving and enhance reliability. However, this model primarily focuses on peak shaving and reliability, neglecting the critical aspect of renewable energy source integration. This limitation restricts its applicability in scenarios where significant RES penetration is a defining characteristic.

Ju et al. [26] propose an innovative micro-energy grid (MEG) structure that integrates a hydrogen energy storage (HES) system with a carbon capture and utilization (CCU) system, collectively termed the HES-CCU-based MEG. This novel configuration introduces a mechanism for interactions between carbon emissions and green certificate equivalents, fostering environmental and economic synergies. A two-stage optimal dispatching framework, consisting of a day-ahead robust dispatching model and a real-time rolling optimization model, is developed to manage uncertainties. Additionally, a benefit allocation method based on entropy and the Shapley value is formulated to equally distribute advantages across system components, including energy conservation, carbon emissions reduction, and renewable energy consumption. Fan et al. [27] present a regional integrated ESS designed for a simulated coastal community in Hong Kong, combining BSSs, compressed air energy storage, and thermal energy storage. The study advances a multi-objective optimization framework that evaluates various energy storage capacities in alignment, economic viability, and environmental performance. This framework emphasizes three distinct energy management strategies, addressing differing priorities for energy storage allocation. Zhang et al. [28] propose a methodology for the optimal allocation of source storage capacity by incorporating integrated demand response (IDR). Their approach begins by establishing the foundational mechanism of the integrated energy system (IES) using an energy hub model that outlines the coupling relationships between system components. An IDR model is then developed to optimize the load curve following real-time market tariff schemes. A confidence interval approach is applied to analyze variability and derive output curves under different confidence levels to address uncertainties in renewable energy generation within the IES. Building on this foundation, a bi-level optimization model is introduced. The upper-level planning model addresses wind and photovoltaic generation uncertainties, aiming to develop an energy storage allocation strategy that minimizes total planning costs. Meanwhile, the lower-level operational optimization model optimizes individual equipment outputs to reduce operational costs while adhering to unit constraints. Kong et al. [29] introduce a multi-objective optimization approach for energy system design, complemented by a multi-criteria evaluation framework for assessing BSS and their suitability within an energy system. Their analysis focuses on grid-connected energy systems integrating wind turbines (WT), photovoltaic (PV) systems, and BSS to effectively meet electricity demands. The evaluation framework incorporates multiple criteria across technical, economic, and environmental dimensions, with total cost and self-sufficiency identified as the primary objective functions.

Many existing studies have not adequately explored the effects of BSS on SC rates, HC of RES, and transformer load rates in μGs, with a particular focus on sodium-sulfur (NaS) and sodium nickel chloride (Na-NiCl₂) battery storage systems (BSS). This research addresses these gaps by presenting a two-stage optimization framework to enhance the operation of grid-connected μGs. In the first stage, optimal allocation decisions for BSS are determined to improve the SCR of RES, increase the HC of these resources, and minimize the overall operational costs of the μG. The second stage builds on the results of the first stage by developing optimal operational strategies for the μG to reduce total operational costs further. The study examines five scenarios, incrementally increasing the number of BSS units from one to five to investigate the impact of quantity and type of BSS on operational efficiency. To solve the optimization problem, the basic open-source nonlinear mixed integer programming (BONMIN) solver in GAMS software is employed. BONMIN’s algorithmic capabilities allow it to effectively manage the mixed-integer nonlinear programming (MINLP) formulation, addressing both discrete and continuous decision variables inherent in this study. This study makes several significant contributions to advancing the integration of BSS and optimizing grid-connected μGs in high-RES penetration scenarios. These contributions are as follows:

1. Optimal BSS allocation strategy: An allocation strategy for BSS is employed, aiming to enhance the SCR of RES. This approach minimizes the total operational costs of μGs, offering a scalable solution for systems with substantial RES integration. The allocation method provides critical insights into BSS’s capacity requirements for maximizing economic and operational efficiency.
2. Economic operation optimization model: A comprehensive economic operation model for μGs is developed in the second phase of the optimization framework. This model formulates strategies to reduce operational expenses by optimizing energy flows, grid interactions, and BSS operations based on the allocation results.
3. HC and transformer loading analysis: The study provides an in-depth evaluation of the impact of BSS integration on the HC of RES and transformer loading. The findings reveal that appropriately placed and sized BSS increase HC and alleviate transformer overload by shifting demand from peak to off-peak hours, delaying infrastructure upgrades.
4. Performance evaluation under varying BSS efficiency and depth of discharge (DoD): The study examines the relationship between BSS efficiency, DoD, and the total operational costs of μGs. Results indicate that improvements in BSS efficiency and higher DoD levels yield significant cost savings and operational performance enhancements, offering practical guidance for technology selection and operation.
5. Systematic scenario analysis: The research demonstrates the influence of BSS quantity and type on μG performance through five distinct scenarios involving incremental increases in BSS. Integrating three Na-NiCl₂ BSS units (Case 3) achieves the highest cost-saving ratio of 32.35%, alongside notable improvements in SC and HC rates, establishing a benchmark for optimal μG configurations.

These contributions provide insights into BSS allocation and operation in μGs, fostering the integration of RES while ensuring economic and technical sustainability.

The structure of this work is organized as follows: Section 2 provides a detailed description of the μG configuration, including its components, layout, and operational characteristics, to establish the foundation for the study. Section 3 focuses on the HC of RES, exploring the factors affecting HC and its role in enhancing μG performance. Section 4 presents the mathematical formulation of the optimization problem, detailing the objective functions, constraints, and parameters used in the two-stage framework and explains the optimization approach, specifically the application of the BONMIN solver. Section 5 presents the simulation results alongside a comprehensive analysis of the findings. Section 6 concludes the work with a summary of the research contributions, key findings, and implications. It also outlines potential directions for future work in optimizing μG operations and integrating advanced BSS solutions.

## 2. Microgrid configuration

**Fig 1:**
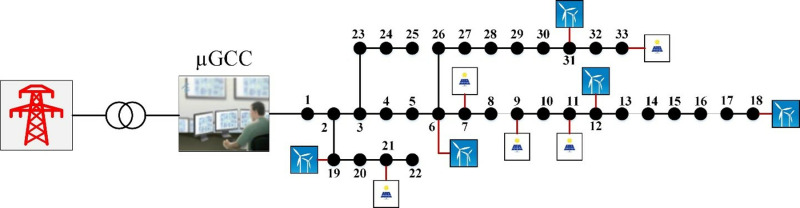
TheμG under study.

The proposed methodology is validated using the modified IEEE 33-bus system, as detailed in [30]. The μG under study incorporates five PV units and five WT units, as depicted in Fig 1. The μG control center (μGCC) plays a central role in optimizing energy flows among the main grid, PV units, and WTs while simultaneously managing the charging and discharging operations of the BSSs. This coordinated management aims to minimize the overall operating costs of the µG. Data for each PV unit and WT, including their capacities and locations, are given in [31] and summarized in Table 1.

**Table 1:** Data for location (bus) and capacity of WTs and PVs.

RES type	PV					WT				
Location (bus)	33	21	11	9	7	31	19	18	12	6
Capacity (MW)	0.6	0.36	0.36	0.36	0.24	1.2	0.96	0.6	0.6	1.2

### 2.1. RES systems

To accurately evaluate the performance of the μG, it is crucial to analyze the factors that impact the output power of its RESs. Electricity generation from PV systems and WTs highly depends on specific environmental conditions. The output power of PV systems is primarily influenced by atmospheric temperature and solar radiation, as outlined in Eq. (1). Similarly, the power output of WTs is determined by the wind speed at a given location, represented as a piecewise function segmented into four distinct regions, as shown in Eq. (2) [31].


$$P_{pv}^{t}=M^{pv}P_{pv}^{rated}\left(\frac{i_{g}}{{si}_{0}}\right)\left(1-{TC}_{coff}\left({TC}_{ambient}-25\right)\right){eff}_{v}{eff}_{R}$$ (1)

$$P_{pv}^{t}=M^{pv}P_{pv}^{rated}\left(\frac{i_{g}}{{si}_{0}}\right)\left(1-{TC}_{coff}\left({TC}_{ambient}-25\right)\right){eff}_{v}{eff}_{R}$$ (2)

where $P_{pv}^{t}$ and $P_{wt}^{t}$ represent the PV and WT output power, respectively. $P_{pv}^{rated}$ and $P_{wt}^{rated}$ represent the PV and WT rated output powers, respectively. $M^{pv}$ represents the units’ number of PV systems. ${si}_{o}$ and $i_{g}$ represent typical solar irradiance at standard testing conditions and the global insolation, respectively. $C_{pv}^{A}$ and $C_{pv}$represent the coefficients for ambient temperature and maximum power output in the PV system, respectively. Also, ${eff}_{R}$ and ${eff}_{v}$ express the PV system’s relative efficiency and the inverter efficiency, respectively. $v_{wt}^{t}$, $v_{wt}^{r}$, $v_{wt}^{CI}$, and $v_{wt}^{CO}$ represent the WT speed, rated speed, cut-in speed, and cut-out speed, respectively.

### 2.2. Operation cost of the main grid

The operating cost of the grid ($C^{M}$) relies on the grid’s output power ($P_{M}^{t}$) and the market energy price ($\beta_{M}^{t}$) in dollars per kilowatt ($/kW) as expressed by Eq. (3) [31].


$$C^{M}=\beta_{M}^{t}\cdot P_{M}^{t}$$ (3)

### 2.3. Storage batteries

Battery storage systems (BSS) come in various types, each with distinct technical characteristics that significantly influence their application in μGs. Common types include lead-acid, lithium-ion, sodium-sulfur (NaS), and nickel-cadmium batteries. Each type exhibits unique features affecting RESs’ HC, PQ assessments, peak load management, grid stability, and energy management in μGs [13]. Among these, NaS batteries are particularly noteworthy due to their advanced performance and adaptability. These batteries employ liquid sodium as the negative electrode and liquid sulfur as the positive electrode, with a solid beta alumina ceramic acting as the electrolyte. NaS batteries are highly regarded for their impressive energy storage capacity, lightweight design, and cost-effectiveness. With efficiencies reaching approximately 95% [32,33], they are well-suited for applications requiring high energy density and reliability. Additionally, their relatively low environmental impact and ability to operate under extreme conditions further enhance their appeal. Similarly, sodium nickel chloride (Na-NiCl₂) represents a sophisticated and versatile energy storage technology. This compound combines sodium, an alkali metal with high reactivity, and nickel chloride, a green crystalline salt widely used in industrial processes like electroplating and catalysis. Sodium’s presence in Na-NiCl₂ enhances the properties of nickel chloride by potentially improving its solubility, stability, and performance in various environments. In the context of energy storage, Na-NiCl₂ offers several advantages, including high energy density and improved electrochemical properties, making it an excellent choice for advanced battery systems. Its role in supporting nickel-based catalysts contributes to chemical processes like hydrogenation and oxidation, highlighting its versatility. These features, combined with the efficiency and reliability of sodium-nickel chemistry, make Na-NiCl₂ batteries a competitive option for grid-scale energy storage and microgrid applications [34,35].

The combination of these battery technologies provides a robust framework for addressing the challenges of integrating RESs into modern power systems, enhancing performance, and driving the transition toward more sustainable energy solutions. However, while both NaS and Na-NiCl₂ batteries are effective for grid-scale energy storage and μGs optimization, they exhibit distinct strengths tailored to different use cases: (i) Efficiency: NaS batteries achieve efficiencies of approximately 95%, slightly outperforming Na-NiCl₂ batteries, which are generally around 90%; (ii) energy density: Na-NiCl₂ batteries offer higher energy density, making them better suited for applications requiring compact storage solutions; (iii) operating conditions: NaS batteries are more robust under extreme temperatures, whereas Na-NiCl₂ batteries excel in chemical stability and versatility; (iv) cost-effectiveness: NaS batteries are typically more cost-effective for large-scale deployment, while Na-NiCl₂ batteries provide higher performance in applications requiring precision and longevity; and (v) lifecycle: Na-NiCl₂ batteries generally exhibit longer lifespans and greater cycle stability, making them more favorable for applications with high cycling requirements. This means that NaS batteries are ideal for large-scale, cost-sensitive applications in severe environments, whereas Na-NiCl₂ batteries provide superior performance in high-density, high-cycle applications [36].

The capital cost of the battery storage system (BSSC) is influenced by its power capacity ($P^{BSS}$) and energy capacity ($E^{BSS}$), as outlined in Eq. (4).


$$BSSC=\left(C^{Power}\cdot P^{BSS}\right)+\left(C^{Energy}\cdot E^{BSS}\right)$$ (4)

where $C^{Energy}$ ($/kWh) and $C^{Power}$ ($/kW) represent the costs related to the energy and power capacities of the battery.

## 3. Renewable energy hosting capacity

**Fig 2:**
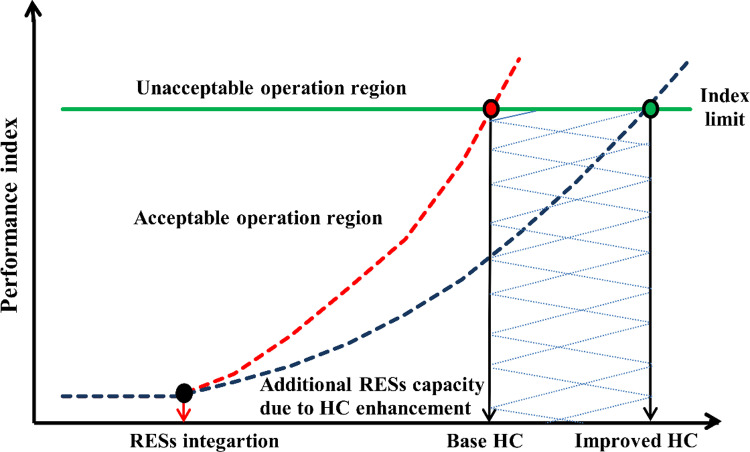
Concept of RES_HC_.

HC for renewable energy represents the upper limit of renewable energy that a power system can accommodate without compromising its reliability or operational performance [37]. This capacity is shaped by multiple factors, including the robustness of the existing infrastructure, the stability of the grid, and the system’s capability to handle variability in energy production from intermittent sources such as solar and wind or even electric vehicles. Conducting comprehensive HC evaluations enables stakeholders to identify opportunities for integrating RESs effectively while preserving PQ and avoiding operational disruptions [38]. Enhancing grid flexibility through advanced energy management and demand response solutions plays a pivotal role in increasing HC. Such measures support the broader adoption of renewable energy, promote a more resilient and efficient energy system, and contribute to reducing dependence on conventional fossil fuel-based power generation [39]. Fig 2 illustrates the concept of RES hosting capacity (*RES*_*HC*_), highlighting that enhancing the HC of the power system allows for greater penetration of RES while meeting operational constraints.

The *RES*_*HC*_ is expressed by the ratio of the total output power injected by RESs ($P_{RES}$) to the apparent power of demand ($S_{D}$), as expressed in Eq. (5) [40].


$$RES_{HC}(\backslash \%)=\frac{P_{RES}}{S_{D}}\cdot 100$$ (5)

The self-consumption rate (*SCR*) of RESs is determined by calculating the ratio of the actual energy output from the RES ($E_{RES}$) to the total rated energy generation capacity of the RES ($E_{RES}^{rated}$), as defined in Eq. (6) [13].


$$SCR=\frac{E_{RES}}{E_{RES}^{rated}}$$ (6)

Maximizing *SCR* ensures that the energy produced from RESs is used efficiently rather than being wasted or subjected to grid constraints. Also, by consuming energy at the source of production, transmission and distribution losses are significantly reduced, improving overall system efficiency. Further details on HC and SCR enhancement are presented in file S1.

## 4. Formulation of the optimization problem

This section presents the development of a two-stage optimization framework aimed at identifying the optimal allocation of BSS and devising the most effective operational strategy for the μG. The framework’s primary goals are to maximize the SCR of RESs, enhance their HC, and minimize the μG’s total operational costs. The mathematical formulation of the problem under investigation is detailed below:

### 4.1. Objective function stages

The objective function is divided into two distinct stages. The first stage focuses on determining the optimal allocation of BSSs to maximize the SCR of all RESs within the μG, as represented by Eq. (7), where Φ(x) denotes the total SCR of all RESs in the μG.


$${f(x)}_{1}=max\Phi (x)=\sum_{t=1}^{T}\left(\frac{E_{E,t}}{E_{E,t}^{R}}\right)$$ (7)

In the second stage, the framework develops optimal operational strategies for the μG, leveraging the results of the first stage to minimize the total operational costs, as expressed in Eq. (8). Here, θ(x) accounts for utility operation costs ($/kWh), generation costs from WTs and PV systems ($/kWh), and the total daily cost of BSS (TBSSC, $/day).


$${f(x)}_{2}=min\theta (x)=\sum_{t=1}^{T}\left(\left(\beta_{M}^{t}\cdot P_{M}^{t}\right)+\left(\beta_{wt}^{t}\cdot P_{wt}^{t}\right)+\left(\beta_{pv}^{t}\cdot P_{pv}^{t}\right)\right)+TBSSC$$ (8)

where $E_{R,t}$ and $E_{E,t}^{R}$ represent the total energy produced by the RESs at hour t and the overall energy generated by RESs at the same hour t, respectively. T indicates the 24-hour horizon and t denotes the time step in hours. $P_{M}^{t}$, $P_{pv}^{t}$, and $P_{wt}^{t}$ refer to the grid output power, WTs’ output power, and the PVs’ output power at hour t, respectively.$\beta_{M}^{t}$ represents the market price of the grid at t, while $\beta_{pv}^{t}$, and $\beta_{wt}^{t}$ represent the bidding prices for PVs and WTs energy ($/kWh) at t, respectively.

The daily cost of the BSS includes both the capital investment (BSSC), expressed in (4) and replacement expenses incurred over the entire project lifespan. To calculate the number of BSS replacements required over the project’s lifespan, the total number of cycles performed by the BSS (${BSS}_{F}$) is first determined using Eqs. (9) and (10), which define the battery’s operational lifetime. Subsequently, Eq. (11) is applied to estimate the battery lifespan (${Life}_{BSS}$) based on the battery’s life cycle capacity (${BSS}_{LC}$) and the total number of cycles ($B_{cycles}$) [9].


$$N_{BSS}(t,i)=\left(m_{b(t)}-m_{b(t-1)}\right)m_{b(t)},\forall t\in T,\forall i\in D$$ (9)

$${BSS}_{F}=\sum_{i=1}^{D}\sum_{t=1}^{T}N_{BSS}(t,i)$$ (10)

$${Life}_{BSS}=\frac{{BSS}_{LC}}{{BSS}_{F}}$$ (11)

In this formulation, $N_{BSS}(t,i)$ serves as an indicator of the BSS’s operational cycles, expressed as a function of *t* (hours) and *i* (days). Here, *D* represents the to*t*al number of working days in a year, set to 365 for this study, with *i* indicating a specific working day. The variable $m_{b(t)}$ is a binary indicator that denotes the operational status of the BSS at *t* and *i*. Specifically, $m_{b}=0$ indicates that the BSS is in discharging mode while $m_{b}=1$ signifies that the BSS is in charging mode. This dis*t*inction enables precise tracking and optimization of the BSS’s charging and discharging behavior throughout its operation. Consequently, the total number of battery replacements (${RF}_{BSS}$) required over the project’s lifespan (*Y*) is determined using the formulation provided in Eq. (12).


$${RF}_{BSS}=\frac{Y}{{Life}_{BSS}}$$ (12)

Accordingly, the total daily cost of the battery storage system (TBSSC) ($/day) is formulated in Eq. (13), considering the interest rate (*r*) associated with funding the BSS.


$$TBSSC=\frac{1}{D\cdot Y}\left(\frac{r(1+r{)}^{Y}}{(1+r{)}^{Y}-1}\cdot BSSC\cdot {RF}_{BSS}\right)$$ (13)

### 4.2. Optimization problem constraints

Operational constraints must be incorporated to ensure the accuracy and feasibility of the solution are given as follows:

#### 4.2.1 Limits of the output power of PVs and WTs.

The output power of PV and WT systems must adhere to their specified minimum and maximum limits, as defined in Eqs. (14) and (15).


$$P_{pv}^{t,min}\leq P_{pv}^{t}\leq P_{pv}^{t,max},\forall t$$ (14)

$$P_{wt}^{t,min}\leq P_{wt}^{t}\leq P_{wt}^{t,max},\forall t$$ (15)

where, $P_{pv}^{t,min}$ and $P_{pv}^{t,max}$ denote the minimum and maximum output power of PVs, respectively, while $P_{wt}^{t,min}$ and $P_{wt}^{t,max}$ represent the minimum and maximum output power of WTs, respectively.

#### 4.2.2 Limits to ensure the balance of the active power flow.

The total power generated by the utility, WT, PV systems, and the power either discharged from the BSS ($P_{BSS}^{DCG,t}$) or charged into the BSS ($P_{BSS}^{CG,t}$) must balance the overall power demand ($P_{D}^{t}$) while accounting for total power losses ($P_{Loss}^{L,t}$), as specified in Eq. (16).


$$P_{wt}^{t}+P_{pv}^{t}+P_{M}^{t}+P_{BSS}^{DCG,t}=P_{D}^{t}+P_{BSS}^{CG,t}+\sum_{L=1}^{NL}P_{Loss}^{L,t}\forall t\in T$$ (16)

where $P_{Loss}^{L,t}$ and NL denote the power losses of the μG at t of the $L^{th}$ line and the number of lines.

#### 4.2.3 Voltage limits.

The root-mean-square (RMS) voltage at each bus must remain within an acceptable range, which, for the purposes of this study, is set between 0.95 and 1.05 per unit (p.u.), as in (17).


$$V_{B}^{min,t}\leq V_{B}^{t}\leq V_{B}^{max,t}$$ (17)

where $V_{B}^{t}$, $V_{B}^{min,t}$, and $V_{B}^{max,t}$ represent the voltage bus, minimum, and maximum voltage boundaries at *t*.

#### 4.2.4 Thermal capacity of the lines.

The thermal limit specified in Eq. (18) restricts the current flow among the μG’s branches.


$${CR}_{L}^{RMS}\leq {TI}_{L,max}^{RMS}$$ (18)

${CR}_{L}^{RMS}$ represents the RMS current flowing through the line while ${TI}_{L,\max}^{RMS}$ denotes the maximum current capacity of the line.

#### 4.2.5 Battery storage limits.

In the problem formulation, it is essential to account for the BSS constraints. Eqs. (19) and (20) express the restrictions on the charging power of the BSS ($P_{BSS}^{CG,t}$) concerning the maximum charging capacity limit ($P_{BSS,max}^{CG,t}$) and on the discharging power of the BSS ($P_{BSS}^{DCG,t}$) concerning the maximum discharging capacity limit ($P_{BSS,max}^{DCG,t}$).


$$P_{BSS}^{CG,t}\leq P_{BSS,max}^{CG,t},\forall t\leq T$$ (19)

$$P_{BSS}^{DCG,t}\leq P_{BSS,max}^{DCG,t},\forall t\leq T$$ (20)

The state of charge (*SoC*) of the BSS, denoted as ${SoC}_{BSS}^{t}$, must remain within predefined upper (${SoC}_{BSS}^{t,max}$) and lower (${SoC}_{BSS}^{t,min}$) limits, as specified in Eq. (21). The current SoC (${SoC}_{BSS}^{t}$) is dependent on the *SoC* from the previous time step (${SoC}_{BSS}^{t-1}$) and the amount of energy discharged or charged at the current time *h*, considering the BSS efficiency ($\eta^{BSS}$), as described in Eq. (21). Additionally, the initial SoC (${SoC}_{BSS}^{in}$) at the start of the operation (t=1) is also defined in Eq. (22).Δt represents the length of the period, usually one hour.


$${SoC}_{BSS}^{t,min}\leq {SoC}_{BSS}^{t}\leq {SoC}_{BSS}^{t,max},\forall t\leq T$$ (21)

$${SoC}_{BSS}^{t}=\left\{ \begin{array}{c} {SoC}_{BSS}^{in}+\Delta t\eta^{BSS}P_{BSS}^{CG,t}-\Delta tP_{BSS}^{CG,t},t=1\\ {SoC}_{BSS}^{t-1}+\Delta t\eta^{BSS}P_{BSS}^{CG,t}-{\Delta tP}_{BSS}^{DCG,t},\forall t\geq 2,t\in T \end{array}\right.$$ (22)

The *SoC* at the end of the horizon should match ${SoC}_{BSS}^{in}$ to ensure that the initial state of charge remains constant, as indicated in Eq. (23). Additionally, the total discharged power should equal the total charged power, adjusted for $\eta^{BSS}$, as expressed in Eq. (24).


$${SoC}_{BSS}^{t}={SoC}_{BSS}^{in},t=T$$ (23)

$$\sum_{t=1}^{T}P_{BSS}^{DCG,t}=\sum_{t=1}^{T}P_{BSS}^{CG,t}\cdot \eta^{BSS}$$ (24)

### 4.3. BONMIN solver

Mixed-integer nonlinear programming (MINLP) problems combine the complexity of integer decision variables with the intricacies of nonlinear relationships, making solving them challenging. Basic open-source nonlinear mixed integer programming (BONMIN), developed as part of the COIN-OR project, is a robust solver specifically designed for tackling convex MINLPs [41]. It is implemented within the general algebraic modeling system (GAMS), offering an efficient platform for addressing optimization problems in energy systems, including those involving µGs and renewable energy integration [41,42].

BONMIN employs a variety of algorithms tailored for MINLP problems, including:

- Branch-and-bound: A fundamental algorithm that iteratively solves nonlinear programming (NLP) subproblems and branches on integer variables.
- Outer approximation: Combines NLP subproblem solutions with mixed-integer programming relaxations to refine solutions iteratively.
- Hybrid algorithms: Merges features of branch-and-bound and outer approximation, offering flexibility for solving problems with varying complexities.
- Extended cutting plane: Incorporates linear approximations for constraint functions inspired by cutting-plane techniques.
- Iterative feasibility pump: Focuses on guiding infeasible solutions toward feasibility through iterative improvements.

These algorithms strengthen the convexity of the objective function and constraints, ensuring efficient convergence to globally optimal or near-optimal solutions for a wide range of applications [41,42] in various domains:

1. Energy systems optimization: BONMIN excels in solving MINLPs for µG design, energy storage allocation, and renewable energy HC optimization. Its ability to handle nonlinearities and integrality makes it ideal for balancing operational costs, resource constraints, and system reliability.
2. Infrastructure planning: It is widely used for planning distributed generation (DG) and storage systems, determining optimal placement and sizing to enhance grid stability and reduce losses.
3. Coordination of devices: BONMIN effectively coordinates distributed energy resources and flexible devices such as flexible alternating current transmission systems (FACTS), improving voltage profiles and reducing active power losses in modern distribution networks [42].

Compared to metaheuristic methods, BONMIN offers several advantages: (i) deterministic solutions: unlike metaheuristic approaches, which may yield varying solutions depending on initial conditions, BONMIN provides consistent, reproducible results; scalability: BONMIN efficiently handles large-scale problems, incorporating numerous variables and constraints; and integration with GAMS: the solver’s seamless integration with GAMS simplifies model formulation, allowing for precise representation of complex energy systems [41].

For more details, readers can refer to [41], which discusses the application of BONMIN in energy-related optimization scenarios, specifically focusing on its implementation in RESs and µG operations, and [42], which provides a foundational overview of BONMIN and its role in solving convex MINLP problems and detailing BONMIN’s algorithmic approaches and its integration with modeling systems like GAMS [19]. The main part of the code is presented in file S2.

## 5. Simulation results and their analysis

**Fig 3:**
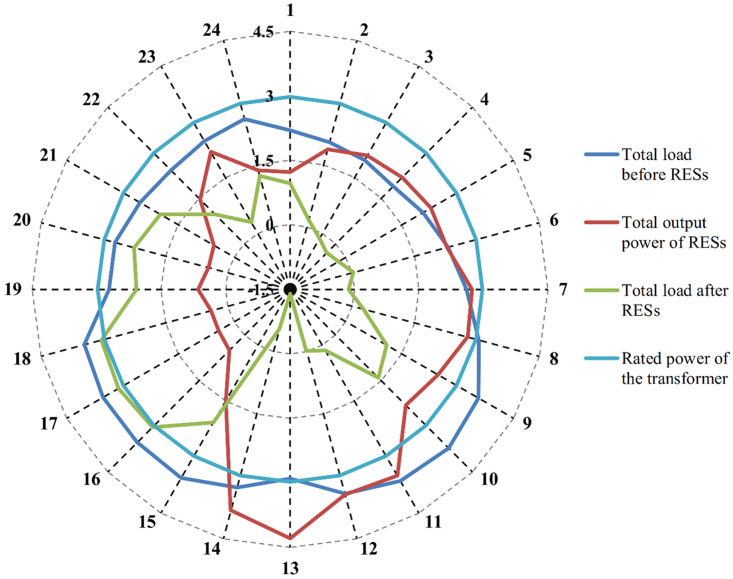
Total output power of RESs, aggregate load, and the rated power of transformer within theµG.

Fig 3 presents the hourly projected output power of all RESs, the aggregated load, the resulting variance between them, and the active power capacity of the transformer for a typical day. The graphical depiction in Fig 3 clearly highlights that an increase in the output power of DGs correlates with a reduction in the main grid’s power contribution. A notable power dynamic reversal occurs when the total output power of the RESs exceeds the aggregate load, particularly during hours 3–5, hour 7, and hours 12–14. Additionally, instances of transformer overloading are observed when the RES output power is low while the aggregated load is high, such as during hour 17. This work initially determines the optimal BSS distribution to improve the *SCR* of all RESs and minimize the total operational costs of the µG. Subsequently, it outlines the standard operational strategy for the BSS within the µG to ensure an accurate reflection of practical operation conditions.

For the µG illustrated in Fig 1, Table 2 summarizes the bid values for the PV and WT units [43]. Meanwhile, Table 3 provides detailed information on the cost parameters, efficiency metrics, and lifetime characteristics of the batteries employed in this study [9,34].

**Table 2:** Bids of PV and WT units.

RES type	Bids ($/kWh)
PV	2.8
WT	1.72

**Table 3:** Cost breakdown, efficiency, and lifecycle of batteries utilized in this work.

Battery	Capital power cost ($/kW)	Capital energy cost ($/kWh)	Efficiency (%)	Lifecycle	Lifetime (years)
NaS	350	300	95	4500	15
Na-NiCl_2_	200	100	90	5500	15

**Fig 4:**
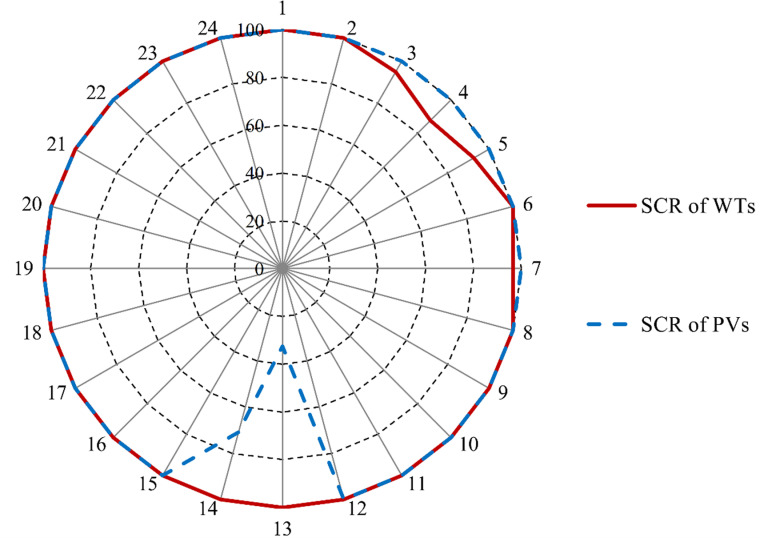
The *SCR* of the PV and WT with no BSS.

Fig 4 provides an hourly analysis of the *SCR* for RES integration in the absence of BSS, representing the base case scenario. Notably, the *SCR* values for WT and PV systems do not consistently reach 100% throughout the day. For example, the *SCR* of WT ranges between 87.7% and 95.0% during hours 3–5, with a peak of 96.6% at hour 7. Similarly, the *SCR* of PV fluctuates between 32.4% and 70.9% during hours 13–14. To enhance the cost efficiency of electricity generation, the µG aims to maintain a consistent 100% *SCR* for both WT and PV systems across all hours of operation.

This study begins by determining the optimal distribution of BSSs to enhance the SCR of interconnected RESs while simultaneously increasing the HC of the µG. Building on the results of this initial phase, the second phase focuses on minimizing the µG’s total operational expenses for a representative evaluation day with the integrated batteries. To analyze the influence of varying BSS quantities on µG performance, five distinct cases are examined:

- Case 1: Incorporation of a single BSS
- Case 2: Incorporation of two BSSs
- Case 3: Incorporation of three BSSs
- Case 4: Incorporation of four BSSs
- Case 5: Incorporation of five BSSs

This multi-scenario analysis provides valuable insights into how increasing the number of BSSs affects the efficiency and operational stability of the µG. Table 4 presents the optimal sizing and placement of BSSs determined using the BONMIN solver for the 5 analyzed cases, aiming to optimize the SCR. The integration of one or more batteries into the µG infrastructure enables the SCR for both WT and PV systems to consistently reach 100% across all diurnal periods. This outcome highlights the significant positive impact of BSSs on enhancing the SCR of RESs, which can lead to substantial cost reductions in µG operations. As a result, such battery solutions become increasingly attractive to both prosumers and µG operators, driving further adoption and optimization of RESs.

**Table 4:** Optimal results of the BSS allocation in the µG.

Number	1	2		3			4				5				
Location (bus)	6	14	29	3	14	30	6	14	24	30	5	6	13	16	26
Power (MW)	2.17	0.64	1.01	1.72	0.559	0.78	0.568	0.5	0.923	0.687	0.429	1.09	0.329	0.211	1.266
Energy (MWh)	13.02	3.84	6.06	10.32	3.354	4.68	3.408	3	5.538	4.122	2.574	6.54	1.974	1.266	4.458

After determining the optimal location, size, and number of BSS, the next step involves establishing the optimal energy management strategy for the µG. This strategy aims to minimize operating costs while evaluating the economic impact of BSS integration on overall system performance. Table 5 summarizes the results, detailing the µG’s operating costs with and without BSS connections. It includes a breakdown of operating costs for the grid, PV system, WTs, individual BSS costs, total daily BSS costs, and µG’s total daily operating costs.

**Table 5:** Optimal results and µG’s total daily operating costs with and without BSSs.

Case	BSS	Cost	BSSC	${RF}_{BSS}$	TBSSC	STBSSC ^*^	${f(x)}_{2}$	Saving
		($/day)	($)		($/day)	($/day)	($/day)	(\%)
No BSS	0	193310.8	-----				193310.8	----
NaS	1	148979.3	4665500	3	1533.8	1533.8	150513.16	22.1
Na-NiCl_2_		149723.38	1736000	3	135.89	135.89	149859.27	22.5
NaS	2	159896.9	1376000	3	452.3	1166.2	161063.1	16.6
			2171500	3	713.9			
Na-NiCl_2_		160805.83	512000	3	40.07			
						103.32	160909.15	16.8
				808000	3	63.24		
NaS	3	129924.2	3698000	3	1215.8	2162.3	132086.5	31.7
			1201850	3	395.1			
			1677000	3	551.4			
Na-NiCl_2_		130568.98	1376000	3	107.71	191.56	130760.54	32.35
				447200	3	35.1		
				624000	3	48.84		
NaS	4	137678.7	1221200	3	401.5	1893.1	139571.8	27.8
			1075000	3	353.4			
			1984450	3	652.4			
			1477050	3	485.6			
Na-NiCl_2_		138965.56	454400	3	35.56	167.7	139133.26	28.02
				400000	3	31.31		
				738400	3	57.8		
				549600	3	43.02		
NaS	5	135957.3	922350	3	303.2	1980.7	137938	28.6
			2343500	3	770.5			
			707350	3	232.6			
			453650	3	149.1			
			1597450	3	525.21			
Na-NiCl_2_		137125.68	343200	3	26.86	175.46	137301.14	28.97
				872000	3	68.25		
				263200	3	20.6		
				168800	3	13.21		
				594400	3	46.52		

*STBSSC denotes the total battery storage system cost ($/day).

In the base case (Case 0), where no BSS is utilized, the total daily operating cost is $193,310.8. This serves as a reference point and incorporates expenses related to µG upgrades and energy losses. Subsequent scenarios explore the inclusion of NaS and Na-NiCl₂ BSS options. Each scenario exhibits distinct cost structures, showcasing the potential savings and economic benefits of integrating these advanced storage solutions into the µG. The total daily cost of the BSS includes capital and replacement costs over the project’s 35-year lifespan, assuming a 0.02 interest rate. By referencing battery life cycles (as detailed in Table 3) and annual cycle counts, the battery’s life expectancy (${Life}_{BSS}$) is calculated to determine replacement needs throughout the project’s duration. Furthermore, the percentage of cost savings in each case is calculated based on the base case’s operating costs.

In Case 1, where NaS BSS is utilized, the daily total operating cost of the µG, including the total battery storage system cost (*STBSSC*), amounts to $150,513.16, reflecting a notable 22.1% cost reduction compared to the reference Case 0. Similarly, in Case 1 with Na-NiCl₂ BSS, the daily total operating cost of the µG is $149,859.27, achieving a slightly higher cost saving of 22.5%. These results underscore the significant cost-effectiveness of incorporating BSS technologies into µG operations.

In Case 2, which involves both NaS and Na-NiCl₂ alternatives, the daily total operating cost is $161,063.1 for NaS, translating to a 16.6% saving relative to Case 0. For Na-NiCl₂, the daily cost is slightly lower at $160,909.15, resulting in a 16.8% saving compared to the reference case.

In Case 3, the NaS-based scenario yields a daily total operating cost of $132,086.5, corresponding to a substantial 31.7% saving compared to Case 0. Meanwhile, with Na-NiCl₂, the cost is further reduced to $130,760.54, delivering the highest saving of 32.35% among all scenarios, highlighting the superior economic advantages of this configuration.

In Case 4, the NaS scenario results in a daily operating cost of $139,571.8, offering a 27.8% saving relative to Case 0. For Na-NiCl₂, the operating cost is $139,133.26, slightly better with a 28.02% saving.

Finally, in Case 5, the NaS-based scenario achieves a daily cost of $137,938, equating to a 28.6% saving compared to the base case. The Na-NiCl₂ scenario delivers slightly better performance, with a daily cost of $137,301.14, reflecting a 28.97% saving.

These comparisons emphasize the cost-effectiveness and operational efficiency gains achievable through the integration of BSS into µG systems. The results clearly demonstrate that the selection of battery storage technology—whether NaS or Na-NiCl₂—significantly impacts the financial outcomes. Na-NiCl₂ consistently outperforms NaS in terms of cost savings across all scenarios, with Case 3 emerging as the most economically advantageous configuration. This analysis highlights the importance of carefully evaluating BSS types and their respective financial implications to optimize energy storage solutions, enhance µG efficiency, and achieve significant cost savings under various operational scenarios. Additionally, the results demonstrate that the total operating cost of the µG is substantially affected by both the number of BSSs connected and the type of BSS implemented. As highlighted in Table 5, Case 3, which integrates three Na-NiCl₂ BSS units, achieves the most significant operating cost-saving ratio of 32.35%. This finding underscores the critical role that optimal BSS configuration and technology selection play in maximizing economic benefits and operational efficiency within µG systems. Besides, the relative comparison, i.e., the savings ratio (Na-NiCl₂/NaS), averaged 1.03 across all cases, highlighting a marginal yet consistent advantage of Na-NiCl₂ over NaS. The cumulative total savings across all cases were $277,014 for NaS and $284,520 for Na-NiCl₂, further emphasizing the economic superiority of Na-NiCl₂ in µG applications.

**Fig 5:**
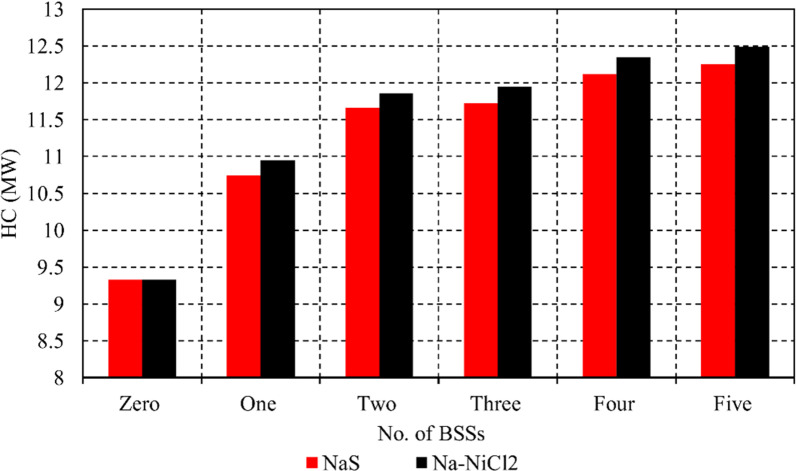
RES_HC_ in the studied µ G in different cases with and without NaS or Na-NiCl2 battery connections.

Fig 5 illustrates the HC values of the examined µG across various cases, both without battery connections and with NaS or Na-NiCl₂ battery connections. For all cases, Na-NiCl₂ provides approximately 2% higher HC than NaS. The data demonstrates a clear correlation between the number of batteries integrated into the system and the enhancement of RES penetration.

In the base case without batteries, RES penetration is 9.33 MW. With the introduction of NaS in Case 1, it increases to 10.75 MW, while Na-NiCl₂ achieves a slightly higher value of 10.95 MW. This upward trend continues in Case 2, where HC values rise to 11.66 MW for NaS and 11.86 MW for Na-NiCl₂. In Case 3, the HC further improves to 11.73 MW with NaS and 11.95 MW with Na-NiCl₂. Similarly, in Case 4, HC reaches 12.12 MW for NaS and 12.35 MW for Na-NiCl₂, and in Case 5, the values peak at 12.25 MW for NaS and 12.49 MW for Na-NiCl₂. The results depicted in Fig 5 clearly highlight a significant enhancement in *RES*_*HC*_ with the integration of BSSs. Notably, the number of BSS units substantially impacts HC performance, with higher numbers of batteries correlating to increased RES penetration. Moreover, the findings reveal that Na-NiCl₂ consistently outperforms NaS in improving HC across all cases, further emphasizing its superior influence on overall system performance. This analysis underscores the importance of selecting the appropriate BSS technology and configuration to optimize µG performance and RES integration.

**Fig 6:**
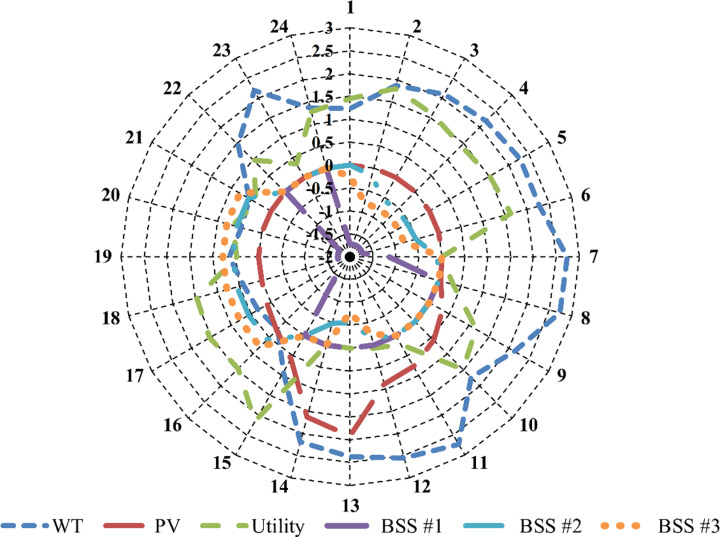
The hourly optimal output powers of the grid, PV, WT, and the BSSs.

Fig 6 depicts the optimal hourly power outputs for the grid, PV systems, WTs, and BSSs in Case 3. The utility curve represents the power exchanged with the grid, where positive values indicate energy drawn from the grid and negative values represent energy fed back into it. The BSS curve illustrates the operational dynamics of the three battery systems, with positive values signifying power discharged to the grid and negative values indicating charging activity. This figure highlights the intricate interactions between RES, grid dependency, and battery management. It underscores the critical role of these components in balancing energy supply and demand, optimizing system performance, and maintaining stability within theµG. The harmonization between grid reliance, RES generation, and BSS operations is vital for achieving efficient energy utilization and ensuring the reliability of the µG system.

**Fig 7:**
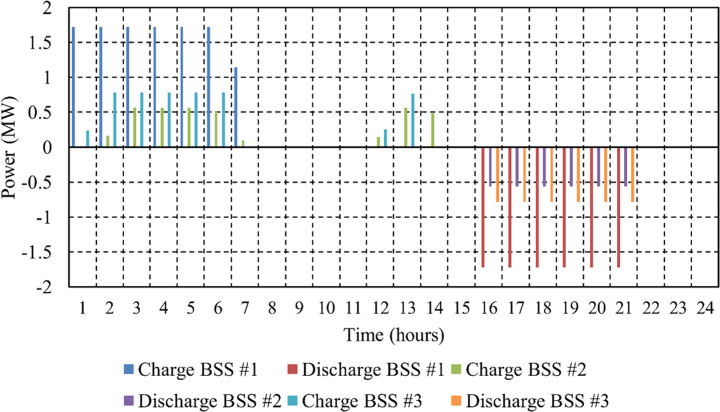
Charge and discharge power of the three Na-NiCl_2_ batteries.

**Fig 8:**
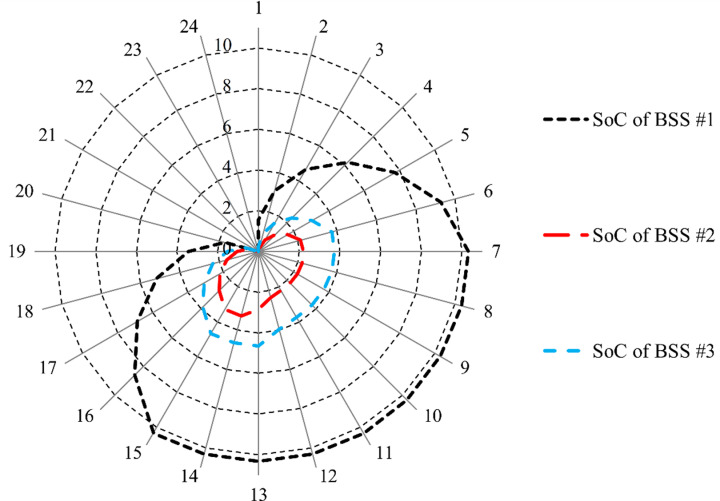
*SoC* of the three Na-NiCl_2_ batteries.

Fig 7 illustrates the charging and discharging power profiles of the three Na-NiCl₂ batteries throughout the day, while Fig 8 depicts the SoC for the same battery storage systems over the same period. Both figures reveal that the batteries are charged during periods of low market prices and reduced overall load, such as from hour 1 to hour 7, ensuring compliance with the technical performance requirements of the µG.

Once fully charged, the batteries begin discharging during periods of high market prices, helping to minimize the µG’s operating costs, as observed between hour 16 and hour 21. This strategic charge-discharge cycle highlights the critical role of BSS in optimizing energy management and enhancing the economic performance of the µG.

**Fig 9:**
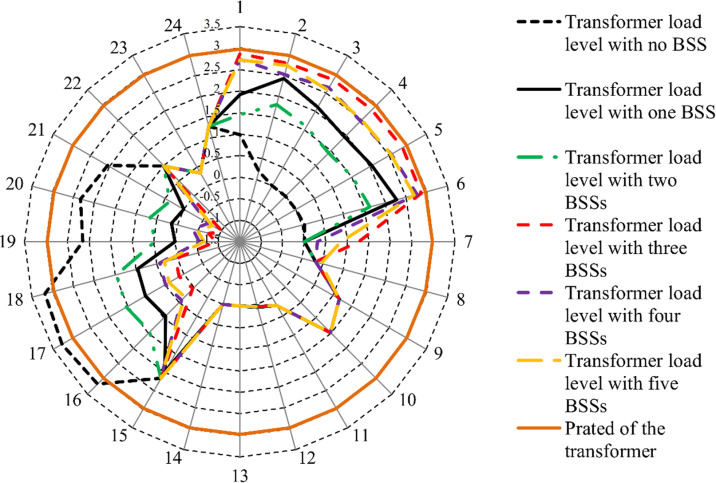
The transformer load level in different cases with and without BSSs.

Transformer overloads can occur when the total output power from RESs is insufficient to meet high overall load demands. In the µG under study, the transformer has a rated capacity of 3500 kVA and a corresponding rated power of 2976 kW. Fig 9 compares the transformer load rates with and without integrating Na-NiCl₂ BSSs.

The figure reveals that, in the absence of Na-NiCl₂, the transformer experiences overload conditions during hours 8–11 and 15–18, exceeding its rated power. However, with the integration of Na-NiCl₂, the transformer load rate remains consistently below its rated power throughout the day. This demonstrates the effectiveness of Na-NiCl₂ in alleviating transformer loading by redistributing demand from peak to off-peak hours. By reducing peak load pressures, Na-NiCl₂ enhances system loadability and delays the need for costly transformer capacity reinforcement, providing operational and economic benefits to the µG.

**Fig 10:**
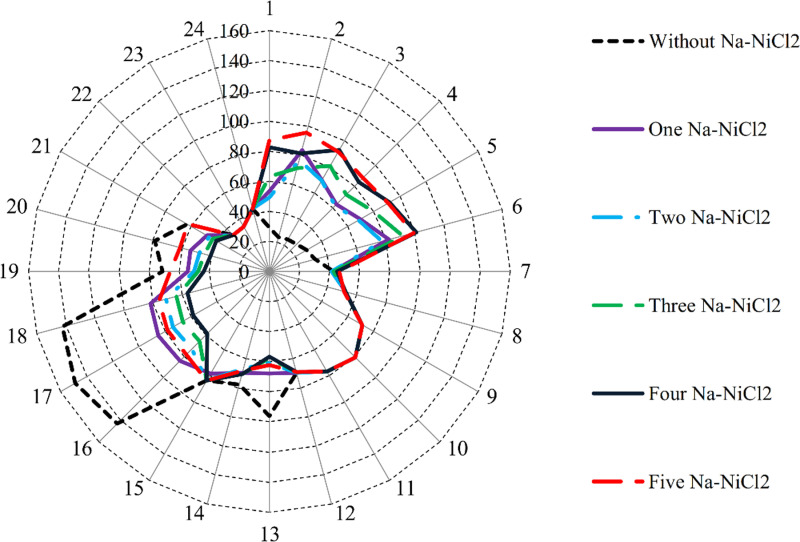
Hourly power losses in the investigated cases with and without BSSs.

Figs 7 and 8 reveal that the BSSs charge during periods of low market prices and reduced load demand, typically in the early hours of the day. However, as depicted in Fig 10, this charging activity increases power losses within the µG during these periods compared to the base case. Interestingly, the power losses remain consistent across all examined cases between hours 8 and 12, reflecting the SoC of the Na-NiCl₂ batteries during this time frame. A notable reduction in power losses is observed between hours 15 and 19, coinciding with the discharge of the Na-NiCl₂ batteries. This means that integrating BSSs decreases the total daily power losses of the µG, from 1571.7 kW in the base case to 1554.58 kW with the inclusion of Na-NiCl₂. This highlights the beneficial role of Na-NiCl₂ in reducing daily power losses, particularly during discharge periods. However, it is also evident that the charging behavior of Na-NiCl₂ can adversely affect power losses during certain periods, necessitating careful management of charging and discharging schedules to optimize µG performance.

**Fig 11:**
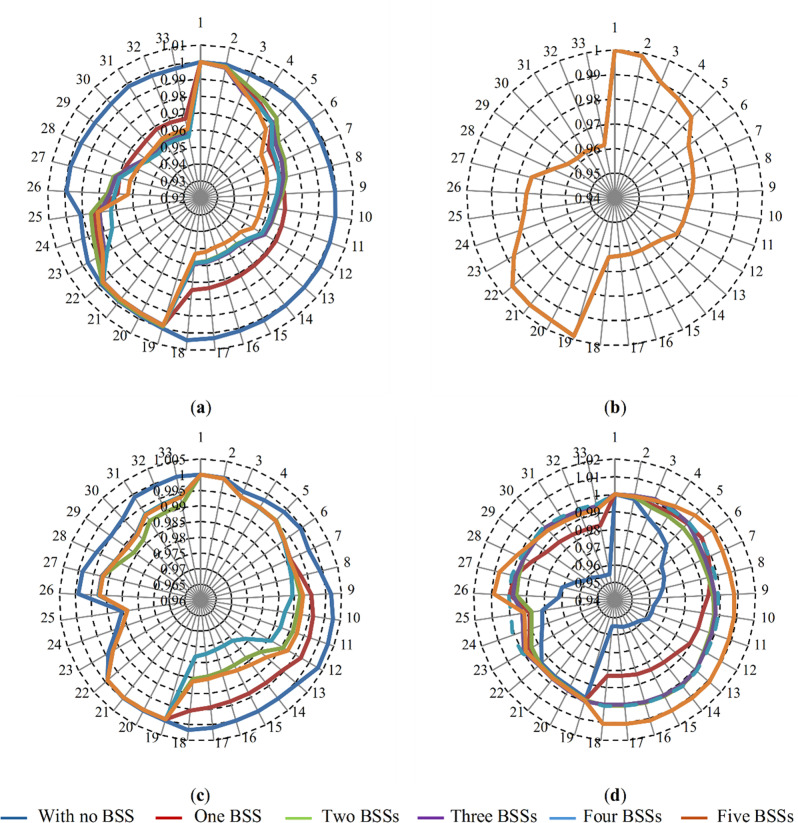
The voltage profile of the µG at four durations at the: (a) 4^th^; (b) 10^th^; (c) 14^th^; and (d) 21^st^ hour.

Fig 11 illustrates the voltage profiles of the µG buses under four distinct loading conditions throughout the day: at hour 4 (51% loading) in Fig 11(a), hour 10 (100% loading) in Fig 11(b), hour 14 (88% loading) in Fig 11(c), and hour 21 (68% loading) in Fig 11(d). In Fig 11(a), under light loading conditions at hour 4, the voltage profile for the base case (without BSS) remains close to 1 per unit across all buses. With the integration of BSSs, the batteries charge during this period due to low market prices, resulting in a slight dip in the voltage profile compared to the base case. However, the voltage levels remain well within acceptable limits. In Fig 11(b), at hour 10 (peak loading), the voltage profile remains consistent across all scenarios, as the SoC of the batteries does not change during this period. Fig 11(c) demonstrates a decline in the voltage profile at hour 14, which is attributed to the charging activity of the BSSs during this time. Conversely, Fig 11(d) shows an improvement in the voltage profile at hour 21, where the BSSs discharge, boosting the voltage levels beyond those observed in the base case. These results highlight the impact of energy storage on the µG’s voltage profile. While charging can slightly lower the voltage during certain hours, discharging BSSs significantly enhances the voltage profile during periods of higher demand, demonstrating their ability to improve grid performance.

**Fig 12:**
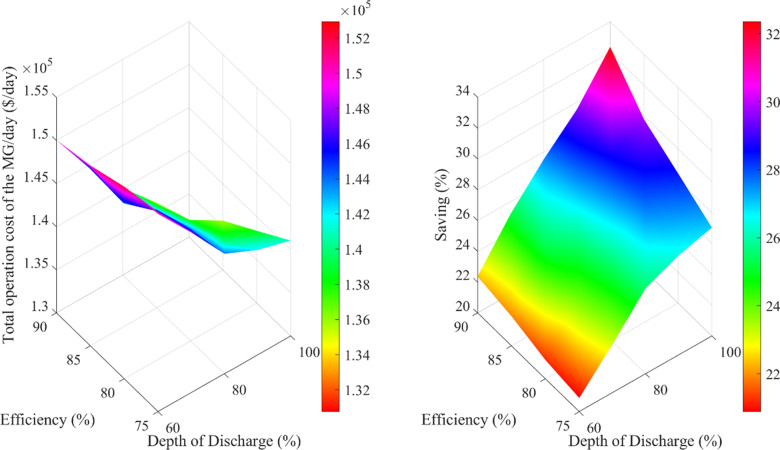
Total operation cost and saving rate variations with efficiency and DoD of Na-NiCl_2_ battery.

Fig 12 depicts the relationship between the total operational cost of the µG and the savings rate as a function of the efficiency and DoD of Na-NiCl₂ batteries.

The figure demonstrates that increasing efficiency from 75% to 90% leads to a notable reduction in the total operational cost, highlighting an inverse relationship between efficiency and cost. For instance, at 75% efficiency with a DoD of 100%, the total operational cost is approximately $141,057, whereas it decreases to around $130,760 at 90% efficiency under the same DoD conditions. Additionally, the savings rate positively correlates with efficiency, indicating that higher operational efficiency results in greater financial benefits. The figure also reveals the DoD’s significant impact on operational cost and savings rate. In general, operational costs are higher at a DoD of 60% compared to a DoD of 100% for the same efficiency levels. These findings underscore the importance of optimizing efficiency and DoD to minimize costs and maximize the economic advantages of integrating Na-NiCl₂ batteries into the µG.

## 6. Conclusions

This work introduces a two-stage optimization framework to enhance the performance of grid-connected µGs by optimizing the integration and operation of BSSs. The first stage focuses on determining the optimal allocation of BSS to maximize the SCR of RESs, increase HC, and minimize total operational costs. The second stage builds upon these results to devise optimal operational strategies that further reduce µG operating expenses. The key contributions of this study are as follows:

1. Proposing a strategic allocation model for BSS that enhances the *SCR* of RESs and minimizes operational costs in microgrids with high-RES penetration.
2. Developing an economic operation optimization model for µG s, incorporating findings from the allocation stage to achieve additional cost reductions.
3. Evaluating the impact of BSS on HC and transformer loading.
4. Analyzing the relationship between BSS efficiency, DoD, and total operational cost savings.

Using the BONMIN solver, the study effectively addressed the nonlinear mixed-integer optimization challenges, ensuring precise and efficient solutions to complex energy management problems. The results indicate that the total operating cost of the µG is significantly influenced by both the number and type of BSS implemented.

Case 3, utilizing three Na-NiCl₂ BSS units, achieved the highest operating cost-saving ratio of 32.35%, demonstrating superior performance in improving HC and reducing transformer loading. Notably, Na-NiCl₂ batteries outperformed NaS batteries in HC improvement and operational stability by alleviating transformer stress and enabling peak demand shifting. Furthermore, total power losses in the µG were reduced from 1571.7 kW to 1554.58 kW, highlighting the efficiency of BSS integration. The study also observed enhanced voltage profiles during specific hours, further contributing to the µG’s reliability.

To build upon the findings of this study, several future directions are recommended: employing machine learning algorithms or advanced metaheuristic methods could complement or enhance the performance of solvers like BONMIN in addressing larger, more complex µG systems and exploration of alternative energy storage technologies such as hybrid BSS solutions, hydrogen storage, or supercapacitors, to assess their impact on µG performance, and finally long-term evaluations of BSS technologies, considering lifecycle costs, carbon footprints, and recycling, that would ensure sustainable deployment. These proposed directions aim further to advance the integration of RESs and energy storage systems, contributing to more sustainable and efficient µG operations.

## Supporting information

S1 File HC and SCR enhancement. (7z)

S2 File The main part of the code. (7z)
